# Scinderin is a potential prognostic biomarker and correlated with immunological regulation: from pan-cancer analysis to liver hepatocellular carcinoma

**DOI:** 10.3389/fimmu.2024.1361657

**Published:** 2024-07-23

**Authors:** Shengyong Zhai, Yuhua Li, Yuanyuan Yang, Wei Lang, Xiaoxia Liu, Kai Liu, Jianjun Qu, Lingyu Zhu

**Affiliations:** ^1^ Department of Gastrointestinal Surgery, Weifang People’s Hospital, The First Affiliated Hospital of Weifang Medical College, Weifang, Shandong, China; ^2^ Department of Nuclear Medicine, Weifang People’s Hospital, The First Affiliated Hospital of Weifang Medical College, Weifang, Shandong, China; ^3^ Department of Anesthesiology, Weifang People’s Hospital, The First Affiliated Hospital of Weifang Medical College, Weifang, Shandong, China; ^4^ Department of Gastroenterology, Weifang People’s Hospital, The First Affiliated Hospital of Weifang Medical College, Weifang, Shandong, China

**Keywords:** Scinderin (SCIN), bioinformatics, pan-cancer analysis, prognosis, liver hepatocellular carcinoma

## Abstract

**Aim:**

This study aimed to systematically dissect the role of Scinderin (SCIN) in tumorigenesis.

**Methods:**

Bioinformatics techniques were employed based on cancer data from TCGA, ENCORI, HPA, GEPIA2, UALCAN, Kaplan-Meier plotter, TIMER, TISIDB, cBioPortal, HCCDB, GeneMANIA and LinkedOmics database. Experiments *in vitro* and *in vivo* were conducted to dissect the role of SCIN in liver hepatocellular carcinoma (LIHC).

**Results:**

Significantly differential expression of SCIN was found in nine types of cancers, including LIHC. Through pan-cancer analysis, the correlations between SCIN expression with prognosis and immune cell infiltration were proven, especially in LIHC, ovarian serous cystadenocarcinoma and lung adenocarcinoma. The highest frequency of alteration in SCIN (6.81%) was seen in patients with uterine corpus endometrial carcinoma, in which “mutation” was the predominant type, with a frequency of about 5.29%; meanwhile, S673F and S381Y were the two most frequent mutation sites. Furthermore, the abnormal expression of SCIN exhibited a strong relationship with immune cell subtypes, immune checkpoint genes, tumor mutation burden, microsatellite instability, neoantigen, molecular subtypes, mismatch repair signatures and DNA methyl-transferase in different cancer types. Through comparative analysis, we discovered that SCIN was dramatically up-regulated in LIHC, and associated with poor survival. Experiments *in vitro* and *in vivo* suggested the knockdown of SCIN could suppress tumor cell proliferation and improve the survival rate partly in animal models.

**Conclusion:**

This study reveals SCIN may be a promising biomarker for prognosis and treatment in certain cancers, especially in LIHC.

## 1 Introduction

Cancer is predicted to rank as the most important obstacle to improve life expectancy in the 21st century (1). Tumor is a complex microenvironment composed of malignant tumor cells, which brings difficulties to the implementation of tumor therapy (2). Given the prevalence of tumors and the complexity of tumorigenesis, it is of great significance to explore the expression of pan-cancer-related genes and assess their levels for clinical treatment and prognostic prediction (3).

Scinderin (SCIN) belonging to the gelsolin family of actin-severing proteins, which is reported to play a regulatory role in the actin cytoskeleton Ca2+-dependently (4). By controlling F-actin dynamics and cytoskeleton underneath the plasma membrane, SCIN plays a key role in vesicle translocation and endocytosis and is upregulated in a series of tumors, such as breast, colon, liver, lung, prostate, and stomach tumors (5). In gastric cancer, the overexpression of SCIN is associated with poor overall survival rate (6), and it changes significantly in stages I and IV, stage II and IV, and stage III and IV (7), which may promote the progression of gastric cancer by regulating STAT3 and NF-κB signaling (8). SCIN not only promotes the survival of prostate cancer cell by stabilizing EGFR and MEK/ERK signals (9), but also enhances the proliferation of lung cancer cell (10), and exerts as a functional apoptosis regulator in the hepatocellular carcinogenesis (11).

However, the systematic evidences on the relationship between SCIN and pan-cancer are rare. Therefore, we analyzed the data retrieved from The Cancer Genome Atlas (TCGA) and Genotypic-Tissue Expression (GTEx) databases to conduct a pan-cancer analysis of SCIN in various tumors. Meanwhile, a set of factors, such as expression level, survival status, DNA mutation and genomic methylation were considered to explore the potential role of SCIN in the mechanism of the tumorigenesis of various cancers. The results showed that SCIN may be an effective biomarker, which provides a new perspective for further understanding the mechanism of various cancers.

## 2 Methods

### 2.1 Gene expression analysis

The expression patterns of SCIN in 33 types of tumors and para-cancerous controls were analyzed using Sangerbox tools (http://vip.sangerbox.com/) based on TCGA database. The gene expression data of normal tissues were retrieved from the GTEx database. The differential expression of SCIN in cancers was verified in online ENCORI database (https://starbase.sysu.edu.cn/). The correlations of the SCIN expression with tumor pathological stage and lymph node metastasis were investigated using “Pathological Stage Plot” module of GEPIA2 (12) and the “TCGA analysis” module of UALCAN (13). The immunofluorescence staining images of HEK 293 cancer cells was retrieved to show the subcellular localization of SCIN in cancer cells by “subcellular” module of Human Protein Atlas. HCDDB database was used to verify the expression of SCIN gene in liver hepatocellular carcinoma (LIHC).

### 2.2 Survival analysis

The unified and standardized pan-cancer datasets were downloaded from UCSC database (http://xenabrowser.net/). The correlations between SCIN expression and overall survival (OS), disease-specific survival (DSS) were analyzed and displayed in the forest plots with the implementation of the survival package (version 3.2–7) in R. By the GEPIA2 (http://gepia2.cancer-pku.cn) and Kaplan-Meier Plotter database (http://kmplot.com/analysis/), we re-evaluated the expression of SCIN and the OS (overall survival) rate in various cancers.

### 2.3 Immune infiltration analysis

The score data of six kinds of immune infiltrating cells in 33 kinds of cancers were downloaded from TIMER database. The Sangerbox database (http://vip.sangerbox.com/) was used to analyze the correlation between SCIN expression and immune cell subtypes, immune checkpoint genes (14), and ESTIMATE score (15). The relationship between the expression of SCIN and immune subtypes was achieved based on TISIDB database. For validation, the TCGA pan-cancer datasets were downloaded from UCSC (https://xenabrowser.net/). IOBR R package was used to evaluate the immune score of immune cells in each sample of every tumor type based on MCPCounter method (16). The expression data of SCIN in each sample was extracted and the correlation between immune cell infiltration and SCIN was calculated with corr.test within psych package in R. Results were visualized using ggplot2 package.

### 2.4 Genomic heterogeneity analysis

Tumor mutational burden (TMB), microsatellite instability (MSI), and neoantigen are the important characteristics for tumor microenvironment. The correlations between SCIN expression and TMB, miMSI, neoantigen, mismatch repair signature (MMRs), as well as DNA methyltransferases were analyzed based on SangerBox database (http://sangerbox.com/Tool).

### 2.5 Genetic alteration analysis

The genetic alterations of SCIN in pan-cancer were examined based on cBioPortal online tool and the mutation-related survival analysis was conducted.

### 2.6 Protein–protein interaction network and enrichment analysis of SCIN in LIHC

GeneMANIA (http://genemania.org/) is an online tool for finding the function similar genes of a given gene list and can construct the protein-protein network of the given genes based on the function interactions (17). LinkedOmics is a novel tool for analyzing the large scale cancer genomics (18). In this study, we constructed the interaction network for SCIN by GeneMANIA website. LinkedOmics was used to explore the differentially expressed genes associated with SCIN in LIHC. Gene set enrichment analysis (GSEA) was performed to investigate the significant Kyoto Encyclopedia of Genes and Genomes (KEGG) and hallmark pathways in the high SCIN and low SCIN expression groups. The expression and prognosis of common genes were analyzed by Gene Set Cancer Analysis (GSCA).

### 2.7 Validation of the expression and prognostic value of SCIN in LIHC

The RNA-seq data of 374 LIHC patients and 50 normal controls were downloaded from TCGA database (https://xenabrowser.net). RNA-seq expression dataset of 240 LIHC samples and 205 normal controls were retrieved from ICGC database (https://dcc.icgc.org/). The expression measures of SCIN were extracted and the differential expression between patient and control groups was analyzed with the utilization of Wilcox test. HCCDB is a web-based database for HCC genomes (19). The HCC tumor and adjacent tissues datasets were obtained from HCCDB (http://lifeome.net/database/hccdb/home.html) for SCIN expression validation and prognostic value validation in LIHC. In addition, the gene expression profiling of 221 HCC tissues with complete clinical information were downloaded from GEO database (accession number: GSE14520). The tumor samples were divided into SCIN high and low expression, and the survival analysis performed using log-rank test. Human Protein Atlas (HPA, https://www.proteinatlas.org/) is a widely used database for studying protein expression in human tissues (20). The immunohistochemistry-based map of SCIN protein expression was available from HPA database. To explore the association of SCIN expression with clinicopathologic features, the TCGA-LIHC cohort were divided into SCIN high and low expression group. The clinicopathologic features between high and low expression were compared using chi-square test. The prognostic impact of SCIN expression along with clinicopathologic features was further analyzed by cox regression analysis.

### 2.8 Cell culture

The human hepatocellular carcinoma cell lines (Hep3B, HepG2, and MHCC97H) and the normal human hepatocytes HHL-5 were purchased from ATCC cell bank (Shanghai, China). The hepatocellular carcinoma cells were maintained in Dulbecco’s modified Eagle medium (DMEM) supplemented with 10% (v/v) fetal bovine serum, 100 units/mL penicillin and 100 μg/ml streptomycin. HHL-5 cells were cultured in a 1:1 DMEM/F-12 culture medium with 10% FBS, 100 units/mL penicillin, and 100 μg/mL streptomycin. All the cells were incubated routinely in a humidified atmosphere containing 5% CO_2_ at the constant temperature of 37°C.

### 2.9 Lentivirus construction and cell infection

To knock down the expression of SCIN, a lentivirus-mediated short hairpin RNA (shRNA) method was employed. Briefly, the lentiviral vectors containing sh-SCIN (LV-shSCIN) and its corresponding negative control (LV-shNC) were obtained from GenePharma, Shanghai, China. HepG2 cells were incubated with LV-shSCIN or LV-shNC plasmids for 48 h. Then, the supernatant was replaced with complete culture medium. The transfected cells were selected with blasticidin. The knockdown efficiency of SCIN was determined by RT-qPCR analysis.

### 2.10 Reverse transcription real time PCR assay

The expression of SCIN was measured by qRT-PCR assay. Total RNA was prepared with the use of TRIzol reagent (Invitrogen, Carlsbad, CA). cDNA was synthesized using high-capacity cDNA RT kit (Applied Biosystems, Foster City, CA). PCR was conducted under StepOne Real-Time PCR System as the following condition: 95°C for 15s, and 45 cycles of 95°C for 5s, and 59°C for 35s. The specific primers for SCIN were as follows: SCIN Forward, 5’-ATGGCTTCGGGAAAGTTTATGT-3’, SCIN reverse, CTGTTACTTATGTCTGCTAT. The relative expression was determined by normalizing to GAPDH (Forward, TGACTTCAACAGCGACACCCA and reverse, CACCCTGTTGCTGTAGCCAAA) according to 2−ΔΔCq formula.

### 2.11 Cell viability detection

Cell proliferative ability was evaluated using CCK8 assay kit (Mlbio, Shanghai, China). In brief, cells at logarithmic phase were harvested for cell suspension preparation. Cells (1×10^4^/well) were seeded in 96-well plate and maintained at 37°C in an incubator with 5% CO_2_ overnight. Then, 10 μL CCK8 solution was administrated to cell culture in each well. After 4h-incubation, the absorbance of cell culture was read at a wavelength of 450 nm under a microplate reader (Bio-RAD, USA). Finally, the growth curve of cells in each group was made to observe the changes of cell proliferative ability.

### 2.12 Animal experiments

HepG2 xenograft model is widely used to mimic the human tumor conditions *in vivo*. Experimental models of HCC provide valuable tools to evaluate the new treatment modalities and biologic characteristics. To explore the role of SCIN *in vivo*, we constructed HCC mice model with HepG2 cells. The BALB/c nude mice were purchased from laboratory animal resources, Chinese academy of sciences. The animal care and ethics committee of Weifang People’s Hospital approved this study and the animal experiments were conducted followed by care and use of laboratory animals’ guidelines.

Total 32 male BALB/c mice aged 6 weeks were assigned to four groups (n=8 per group), including control, model, shSCIN and shNC groups. Mice in model, sh-SCIN and shNC group were subjected to subcutaneous injection with HepG2 cells (1×10^6^). Mice in shSCIN and shNC group were injected with 2×10^6^ HepG2 LV-shSCIN cells and HepG2 LV-shNC cells via tail veins, respectively. Tumor volume and body weight were monitored twice a week according to the previous method (21). The survival time of mice was recorded.

### 2.13 Statistical analysis

The same experiment was repeated for at least three times. Data were displayed as mean ± standard deviation and analyzed by Graphpad prism software. A comparison of difference between groups were achieved by unpaired t test and the multi-group comparison was conducted by one-way analysis of variance with Tukey method. When p value < 0.05, the statistically significance was considered.

## 3 Results

### 3.1 SCIN expression in various cancers

**Figure 1:**
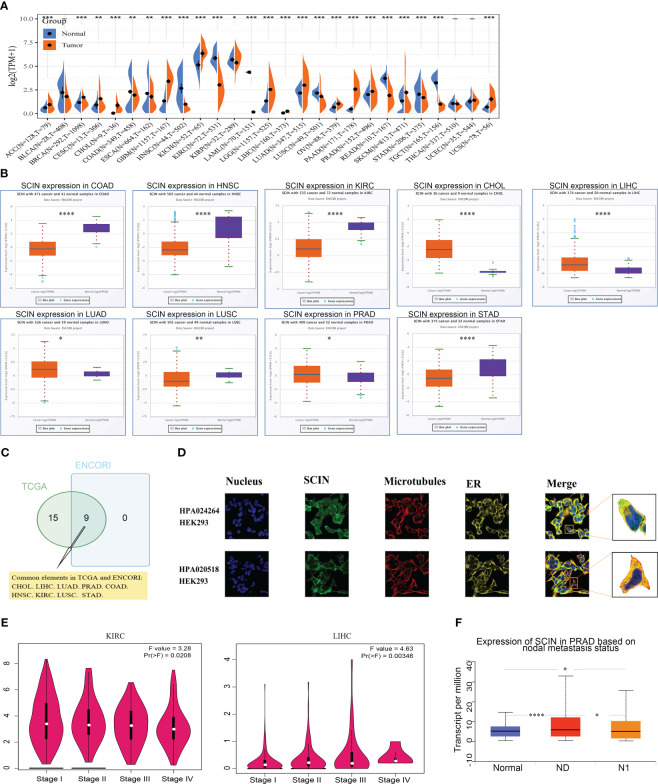
SCIN expression profiles. **(A)** Expression of SCIN in tumor and normal tissues based on TCGA database. **(B)** SCIN expression level in pan-cancer based on ENCORI database. **(C)** Venn plot of the tumor types with differential expression of SCIN based on TCGA and ENCORI database. **(D)** The immunofluorescence images of SCIN protein (HPA020518 and HPA024264), nucleus, endoplasmic reticulum (ER), microtubules and the incorporative images in HEK 293 cell lines. **(E)** SCIN expression in different stages of KIRC and LIHC. **(F)** SCIN expression in PRAD based on metastasis status. *P < 0.05, **P < 0.01, ***P < 0.001, ****P < 0.0001.

Firstly, the expression profiles of SCIN in different tumors and normal counterparts were analyzed based on TCGA and GTEx datasets. As shown in **Figure 1A**, SCIN was overexpressed in ACC, BRCA, CESC, CHOL, GBM, KICH, LGG, LIHC, LUAD, OV, PAAD, PRAD, SKCM, and UCS, while de-expressed in COAD, ESCA, HNSC, KIRC, KIRP, LAML, LUSC, READ, STAD, and TGCT. SCIN expression was further determined in the ENCORI database, which indicated that SCIN expression was significantly elevated in CHOL (P = 5.5e-6), LIHC (P = 2.6e-6), LUAD (P = 0.018), and PRAD (P = 0.019), but down-regulated in COAD (P = 1.0e-32), HNSC (P = 1.1e-23), KIRC (P = 3.7e-38), LUSC (P = 0.0061), and STAD (P = 4.8e-6) (**Figure 1B**). As shown in Venn diagram (**Figure 1C**), nine types of cancers elicited differential expression of SCIN in both TCGA and ENCORI datasets, including CHOL, LIHC, LUAD, PRAD, COAD, HNSC, KIRC, LUSC, and STAD.

Secondly, we found that SCIN proteins included HPA 020518 and HPA 024264 from the Human Protein Atlas (HPA). Immunofluorescence images of cancer cells showed that these proteins were mainly located in the plasma membrane of the HEK 293 cell (**Figure 1D**).

Finally, the expression of SCIN was significantly correlated with the pathological stage of KIRC (P = 0.0208) and LIHC (P = 0.00346) based on “Pathological Stage Plot” module (**Figure 1E**). The “TCGA analysis” module of UALCAN was used to find that SCIN expression was closely associated with lymph node metastasis of PRAD (N0 vs. Normal: P = 2.05e-08,N1 vs. N0: P = 2.50e-02, N1 vs. Normal: P = 3.51e-02) (**Figure 1F**). Taken together, SCIN plays an important role in the recurrence and development of cancers.

### 3.2 Prognostic value of SCIN expression

**Figure 2:**
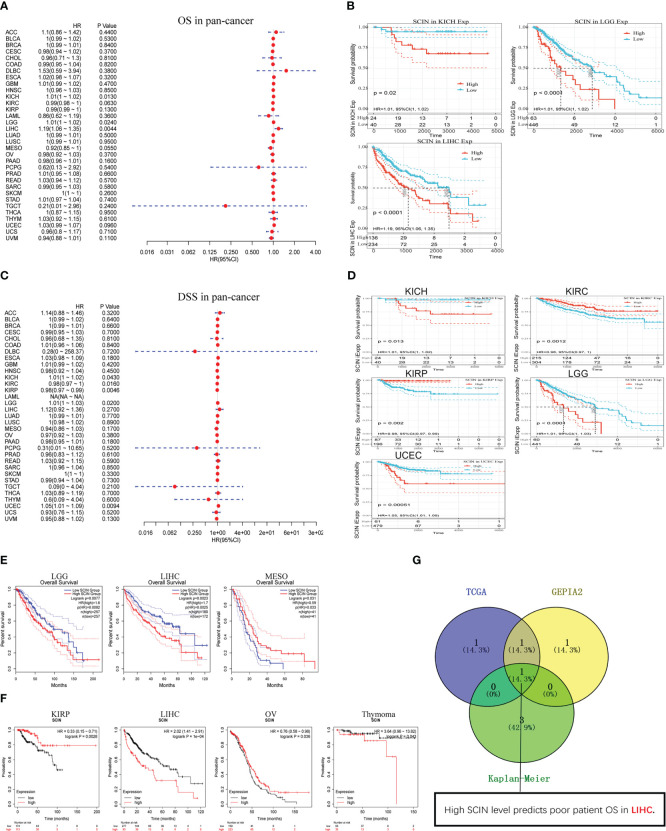
Survival analysis in different types of cancer. SCIN expression and patient prognosis [OS **(A)** and DSS **(C)**] and significant survival curves [OS **(B)** and DSS **(D)**] of different cancers in TCGA database. The OS prognostic value of SCIN in human cancer from GEPIA2 **(E)** and Kaplan-Meier plotter tool **(F)**. **(G)** Comparative analysis of data in TCGA, GEPIA2, and Kaplan-Meier.

Considering that SCIN was maladjusted in multiple types of cancer, we intended to know whether its expression was related to the outcomes of cancer patients. We first analyzed the correlation between SCIN expression with the prognosis of 33 TCGA cancer types. As shown in **Figure 2A**, in KICH (HR = 1.01, 95% CI = 1.00–1.02, P = 0.013), LGG (HR = 1.0 1, 95% CI = 1.00–1.02, P = 0.024), and LIHC (HR = 1.19, 95% CI = 1.06–1.35, P = 0.0044), the high expression of SCIN was associated with poor OS. **Figure 2B** showed a statistically significant survival curve.

In addition, in order to avoid deviations caused by non-cancer events, the disease-specific survival (DSS) was analyzed, as shown in **Figure 2C**. Similar to the results of OS analysis, high SCIN expression of was significantly correlated with the poor DSS of KICH (HR = 1.01, 95% CI = 1.00–1.02, P = 0.043), LGG (HR = 1.01, 95% CI = 1.00–1.03, P = 0.02), and UCEC (HR = 1.05, 95% CI = 1.01–1.09, P = 0.0094). Conversely, low SCIN expression was related to the poor DSS in KIRC (HR = 0.98, 95% CI = 0.97– 1.00, P = 0.016) and KIRP (HR = 0.98, 95% CI = 0.97–0.99, P = 0.0046). The significant survival curve was shown in **Figure 2D**.

Secondly, the prognostic value of SCIN in 33 kinds of cancers was evaluated again based on GEPIA2 and Kaplan-Meier plotter databases. The results of GEPIA2 analysis indicated SCIN high expression predicted the poor prognosis of LGG (P = 0.0077) and LIHC (P = 0.0023), while the prognosis was good in patients with MESO (P = 0.031) (**Figure 2E**). As shown in **Figure 2F**, based on Kaplan-Meier plotter, high expression of SCIN was correlated with poor prognosis of thymoma (P = 0.043) and liver cancer (P = 1e-4). On the contrary, the high expression of SCIN was associated with high OS rate in kidney renal papillary cell carcinoma (P = 0.0028) and ovarian cancer (P = 0.036). As shown in venn diagram (**Figure 2G**), high SCIN expression was verified to be associated with poor overall survival in LIHC based on TCGA, GEPIA2 and Kaplan-Meier plotter databases together. These results clearly determined the significant prognostic value of SCIN expression in many types of cancer, especially in LIHC.

### 3.3 Immune microenvironment, immune checkpoint genes, immune subtypes

**Figure 3:**
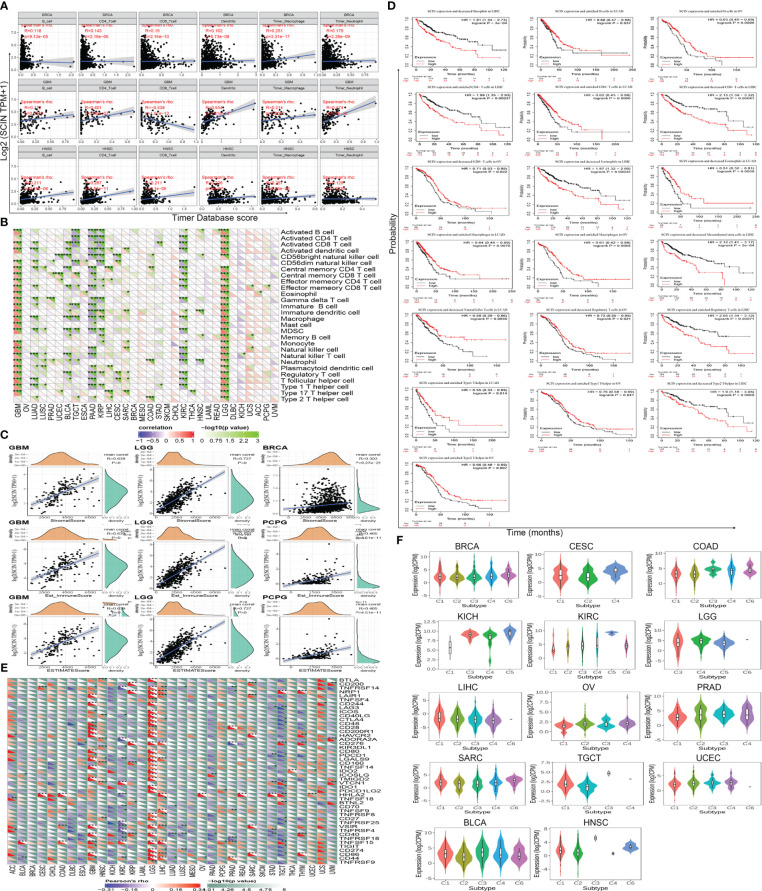
Correlation between SCIN expression level and immune cell infiltration in cancers. Correlations between SCIN expression and **(A)** six immune cell infiltration scores; **(B)** infiltration level of immune cells by xCell in TCGA; **(C)** Top three cancers by ImmuneScore, StromalScore, and ESTIMATEScore, respectively; **(D)** Immune cells score in LIHC, OV and LUAD of OS curves; **(E)** immune checkpoint genes; **(F)** immunity subtypes.

Immune cell infiltration plays a key role in the tumor microenvironment, which regulates the development and deterioration of diverse cancers. The relationship between six kinds of immune infiltration cells and SCIN expression was analyzed based on the TIMER database. As depicted in **Figure 3A**, a significant correlation was found between SCIN expression and CD4+T cells in 20 cancer types, B cells in 21 cancer types, CD8+T cells in 17 cancer types, macrophages in 18 cancer types, neutrophils in 21 cancer types, and dendritic cells in 25 cancer types. Especially in BRCA, GBM and HNSC, the infiltration of CD8+T cells, dendritic cells, macrophages and neutrophils was almost positively correlated with the expression of SCIN. The similar findings were observed in **Supplementary Figure S1**, which verified the accuracy of our findings. Secondly, using the Sangerbox database for further analysis, it was found that SCIN expression was related to the infiltration of 28 subtypes of immune cells in 24 cancers. Especially in LGG, except for neutrophils, the SCIN expression elicited positive correlation with the infiltration of 27 kinds of immune cells (**Figure 3B**). Finally, as shown in **Figure 3C**, SCIN was closely related to Stromal score, Immune Score, and ESTIMATE Score in LGG and GBM, Immune Score and ESTIMATE Score in PCPG, and Stromal score in BRCA. Kaplan-Meier analysis showed that in CESC, KIRC, LUSC, KIRP, SARC, HNSC, STAD, BLCA, BRCA, PADA, and CSCC, especially in LIHC, OV, and LUAD, the abnormal expression of SCIN was closely related to OS. As shown in **Figure 3D**, in the LIHC cohort, the groups with high expression of SCIN and lower scores of basophils (P = 3e-4), CD4+T cells (P = 0.00037), eosinophils (P = 0.00035), mesenchymal stem cells (P = 2e-04), CD8+ cells (P = 0.00041), type 2 T helper cell (P = 0.0069) and higher scores of regulatory T cell (P = 0.00071) showed the worst OS.

We further studied the relationship between SCIN expression and 47 common immune checkpoint genes screened from previous data (14). In GBM, KIRC, LGG, TGCT, and LIHC, SCIN expression was correlated to the expression level of most immune checkpoint genes. In particular, SCIN showed positive correlation with 29 immune checkpoint genes in LGG (**Figure 3E**).

Next, the role of SCIN in human cancer immunity subtypes was dissected based on TISIDB database. There were 6 immune subtypes, including C1 (wound healing), C2 (IFN-gamma dominant), C3 (inflammatory), C4 (lymphocyte depleted), C5 (immunologically quiet), and C6 (TGF-b dominant). As illustrated in **Figure 3F**, SCIN was associated with different immune subtypes in BLCA (P = 7.86e-05), BRCA (P = 2e-03), CESC (P = 3.5e-02), COAD (P = 1.27e-02), HNSC (P = 3.65e-04), KICH (P = 2.16e-02), KIRC (P = 1.46e-02), LGG (P = 3.41e-06), LIHC (P = 4.7e-02), OV (P = 1.33e-04), PRAD (P = 2.31e-02), SARC (P = 2.78e-02), TGCT (P = 1.01e-04), and UCEC (P = 3.28e-03). Thus, SCIN exerts important roles in tumor immunity.

### 3.4 Tumor immunotherapy evaluation

**Figure 4:**
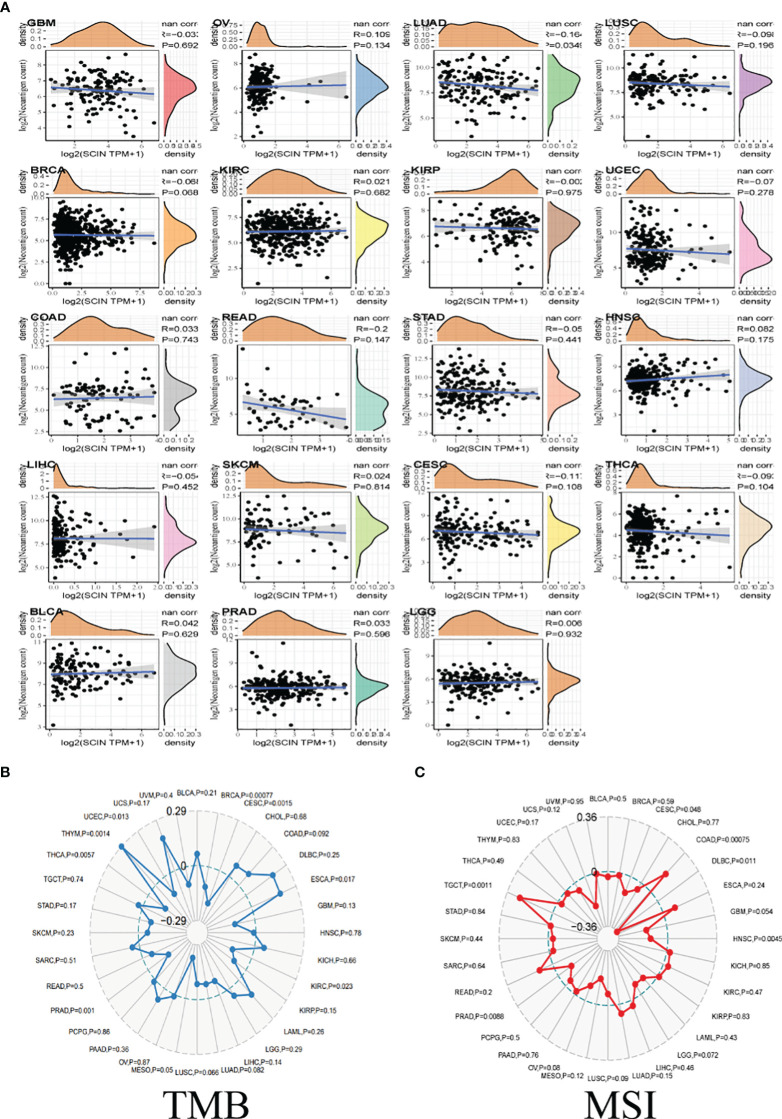
The correlations between SCIN expression and Neoantigen **(A)**, TMB **(B)**, and MSI **(C)**. Spearman’s correlation coefficients are shown above the bar graphs. (Spearman Correlation test, p< 0.05 was considered significant).

TMB, MSI, and neoantigen in the tumor microenvironment were the important biomarkers for tumor immunotherapy, so the correlation between SCIN and TMB, MSI, and neoantigen was analyzed. As shown in **Figure 4A**, there was a reverse correlation between SCIN expression and neoantigen in LUAD, LUSC, BRCA, KIRP, UCEC, READ, STAD, LIHC, CESC, and THCA, but positive correlation with neoantigen in OV, LGG, COAD, HNSC, SKCM, BLCA, PRAD, and KIRC.

As illustrated in **Figure 4B**, in BRCA, CESC, KIRC, PRAD, THCA, and UCEC, the downregulation of SCIN elicited reverse correlation with the decrease of TMB, while SCIN accumulation showed positive correlation with the expression of TMB in ESCA, and THYM.

Further, there were positive association between SCIN and MSI in COAD and TGCT, but negative association with DLBC, HNSC and PRAD (**Figure 4C**). Thus, SCIN may affect anti-tumor immunity by regulating tumor microenvironment.

### 3.5 Molecular subtypes, mutation, MMR signatures, and DNA methylation

**Figure 5:**
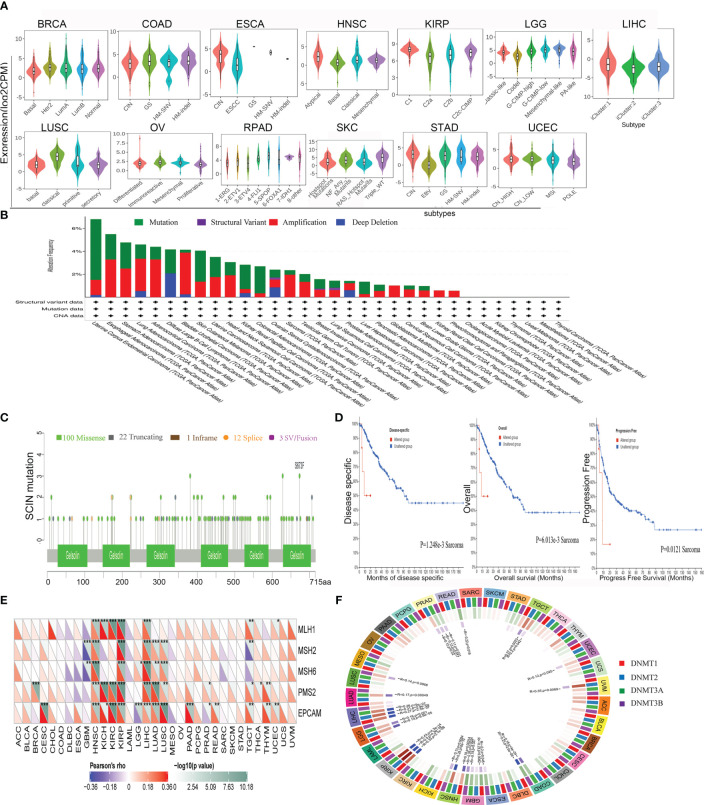
Correlations between SCIN and Genomic heterogeneity analysis in different tumors. **(A)**The relationship between SCIN expression and pan-cancer molecular subtypes. **(B)** The genetic alteration type and frequency of SCIN in various cancers using the cBioPortal tool. **(C)** Mutation diagram of SCIN in different cancer types across protein domains. **(D)** The potential correlation between mutation status and overall, disease-specific and progression-free survival of Sarcoma. The correlations between SCIN and essential genes involved in MMRs **(E)** and DNA methyltransferase **(F)** in multiple cancers. *P < 0.05, **P < 0.01, ***P < 0.001.

The role of SCIN in various tumor molecular subtypes was investigated by the TISIDB database. SCIN was closely associated with molecular subtypes in BRCA (P = 3.53e-08), COAD (P = 1.25e-02), ESCA (P = 9.64e-08), HNSC (P = 3.2e-09), KIRP (P = 1.96e-07), LGG (P = 3.72e-30), LIHC (P = 4.32e-03), LUSC (P = 5.86e-10), OV (P = 2.37e-05), PRAD (P = 1.78e-12), SKCM (P = 3.12e-04), STAD (P = 3.63e-1), and UCEC (P = 3.74e-04) (**Figure 5A**).

It is generally believed that genomic mutation is closely related to tumorigenesis. To understand the genomic mutation of SCIN in cancer, the genetic changes of the SCIN gene in different cancer patients were examined. The highest frequency of alteration in SCIN (5.29%) was seen in UCEC, in which “mutation” was the predominant type, while “amplification” was the predominant type in bladder urothelial cancer, with a frequency of about 3.65% (**Figure 5B**). **Figure 5C** clearly shows that a total of 138 mutations were found between amino acid 0 and 715, including 100 missense, 22 truncating, 1 inframe, 12 splice, and 3 fusion mutations. Among them, S673F and S381Ywere most frequently mutated. The overall survival rate (P = 6.013e-03), disease-specific (P = 1.248e-03), and progression-free (P = 0.0121) survival of Sarcoma patients with unaltered SCIN changes were better, but t no correlation was seen between SCIN gene mutation and prognosis in other tumors (**Figure 5D**).

MMR signatures (MMR) were an intracellular mismatch repair mechanism, in which the dysfunction of key genes was associated with a high rate of somatic mutation. The correlation between SCIN expression and five MMR genes were evaluated. The results revealed SCIN was positively correlated with MMR gene expression in most tumors, especially in HNSC, KIRP, and LIHC, in which the SCIN expression was significantly correlated with MLH1, MSH2, MSH6, PMS2, and EpCAM (**Figure 5E**).

DNA methylation was important in epigenetics, so we analyzed the correlation be-tween SCIN expression and four methylation transferases (DNMT1, DNMT2, DNMT3A, and DNMT3B). SCIN protein expression is associated with one or more methylation transferases in UVM, LUAD, TGCT, LGG, LIHC, KIRP, COAD, GBM, LUSC, UCEC, ESCA, HNSC, and PRAD (**Figure 5F**). The above results show that the changes of the SCIN gene itself may confer the tumorigenesis and malignant progression.

### 3.6 Further validation in LIHC

**Figure 6:**
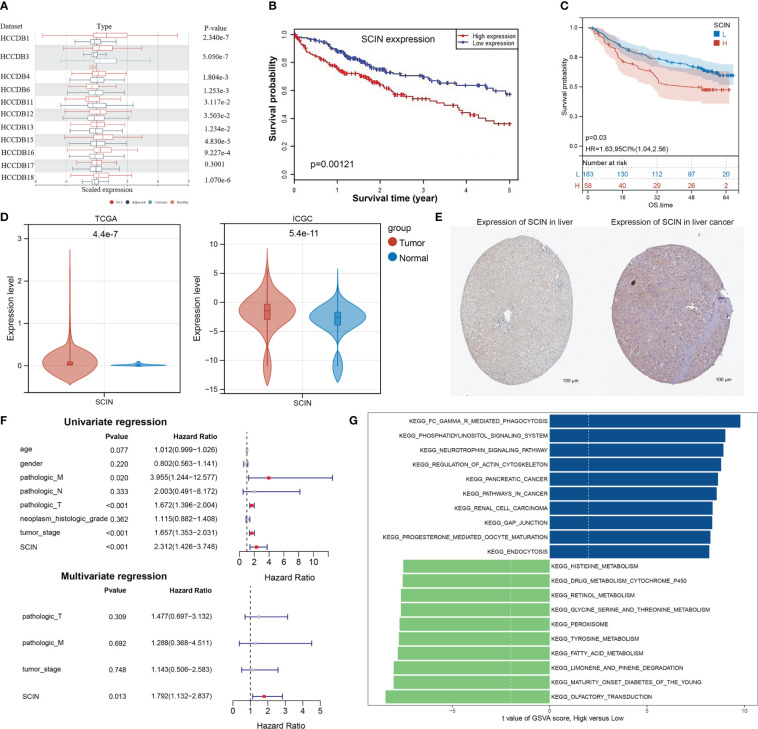
Further Validation SCIN expression in LIHC. **(A)** SCIN expression levels in LIHC and normal tissues based on HCCDB database.**(B)** Overall survival analysis was examined with respect to SCIN expression on the HCCDB database. Correlation of SCIN expression with OS **(C)** Survival analysis with respect to SCIN expression in GSE14520 LIHC cohort **(D)**, differential expression of SCIN in TCGA and ICGC cohorts. **(E)** immunohistochemical images of SCIN expression in liver cancer tissues and normal tissues from Human Protein Atlas (HPA) database. **(F)** Cox regression analysis of the prognostic factors of SCIN along with clinical features. **(G)** The top 10 up- and down-regulated pathways in high SCIN expression group compared with low expression group.

Based on the previous analysis (**Supplementary Figure S2**), the overexpression of SCIN in LIHC was associated with survival, immune cell infiltration, immune checkpoint gene, immune subtype, molecular subtype, MMRs, and DNA methylation. Therefore, we identified LIHC as a representative type of cancer for analysis. Based on 11 datasets (HCCDB 1, 3, 4, 6, 11, 12, 13, 15, 16, 17, and 18) from the HCCDB database, SCIN was upregulated in HCC tissues compared with adjacent normal tissues (**Figure 6A**) and was closely related to poor prognosis of patients (P = 0.00121) (**Figure 6B**). Survival analysis in GSE14520 cohort also showed that LIHC patients with high SCIN expression was associated with poor prognosis (P=0.03, **Figure 6C**). The aberrant overexpression of SCIN in HCC tissues was validated in both TCGA and ICGC cohorts (all P<0.05, **Figure 6D**). Based on the HPA image collection, the protein expression level of SCIN was basically consistent with the trend of gene expression level in tumor and normal control tissues (**Figure 6E**). Then, based on the integrating clinical and pathological data, the relationship between SCIN expression and several clinical characteristics of TCGA-LIHC patients was investigated (**Table 1**). Results showed that SCIN expression was significantly associated with tumor stage (P=0.02578) and pathologic T staging (P=0.0435). The prognostic factors for LIHC was analyzed. Univariate regression analysis revealed that pathologic M, pathologic T staging, tumor stage and high SCIN expression were the risk factors for OS of LIHC patients (all P<0.05, **Figure 6F**). Multivariate regression further indicated that SCIN was an independent prognostic factor for LIHC (P<0.05, **Figure 6F**). GSEA analysis showed that between high and low expression group, cancer related pathways were upregulated in SCIN high risk group compared with low risk group, while the pathways of histidine metabolism, retinol metabolism, glycine serine and threonine metabolism, fatty acid metabolism were downregulated (**Figure 6G**).

**Table 1:** Comparison of the clinicopathological features between SCIN high and low expression groups.

Features	SCIN.High(n=184)	SCIN.Low(n=184)	p value
age			0.09353
<60	74	91	
≥60	110	93	
gender			0.7516
female	70	49	
male	114	135	
neoplasm_histologic_grade			0.5355
G1	25	30	
G2	92	84	
G3	61	59	
G4	4	8	
pathologic_M			0.1907
M0	120	145	
M1	3	0	
pathologic_N			0.5167
N0	115	135	
N1	3	1	
pathologic_T			0.0435
T1	81	101	
T2	53	39	
T3	40	38	
T4	10	3	
tumor_stage			0.02578
stage i	73	99	
stage ii	48	37	
stage iii	43	40	
stage iv	4	0	

Thus, we speculated that SCIN played an important role in tumorigenesis and malignant progression of LIHC.

### 3.7 Co-expressed genes of SCIN in LIHC

**Figure 7:**
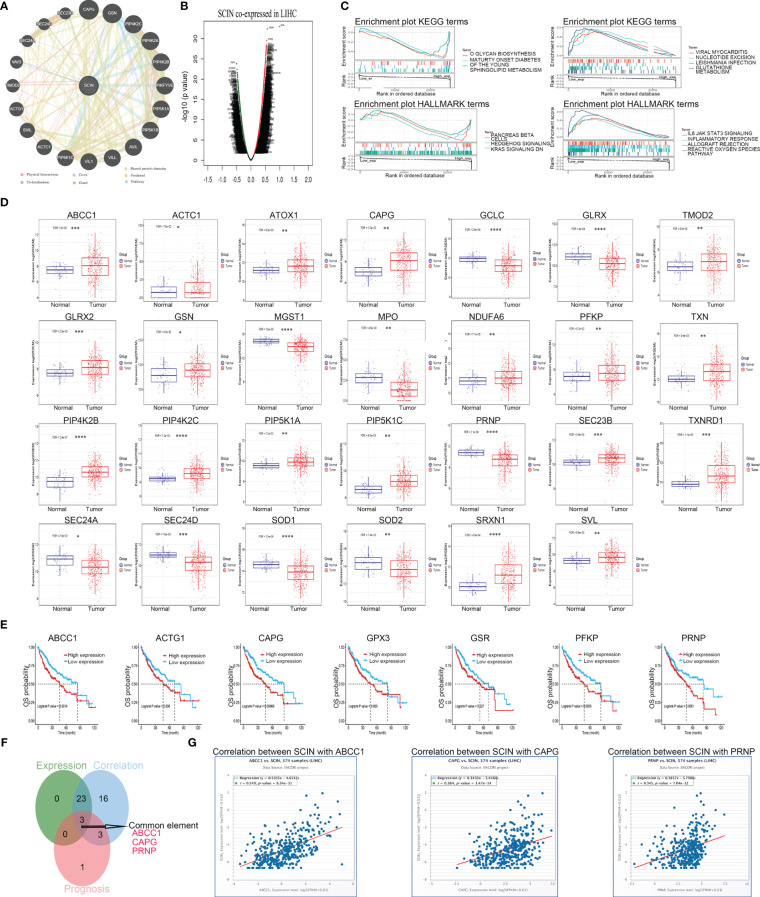
SCI gene affects the occurrence and development of LIHC through some possible genes. **(A)** PPI network for SCIN was constructed in GeneMANIA. **(B)** The co-expressed genes of SCIN in LIHC obtained from LinkedOmics were shown as a volcano plot. **(C)** GSEA for samples with high SCIN expression and low expression. Only gene sets with |NES|>1, P < 0.05 and FDR < 0.25 were considered significant. GSCA database was utilized for analyzing the expression **(D)** and prognosis **(E)** of the core genes in LIHC. **(F)** Data comparison was shown as Venn diagrams. SCIN might play a role in liver cancer through ABCC1, CAPG, and PRNP. **(G)** the co-expression between SCIN and ABCC1, CAPG, PRNP in liver cancer was verified in the GSCA database. *P < 0.05, **P < 0.01, ***P < 0.001, ****P < 0.0001.

To investigate the potential mechanism or possible signaling pathway involved with SCIN in carcinogenesis, we first constructed SCIN’s PPI network using the GeneMANIA database, and the result is shown in **Figure 7A**.

Secondly, co-expressed genes of SCIN in LIHC were analyzed based on LinkedOmics database, and the differentially expressed genes were shown by volcanic map (**Figure 7B**). After GSEA analysis of these genes (**Figure 7C**), HALLMARK enrichment suggested the genes in SCIN low expressed group were mainly related to the reactive oxygen species pathway (FDR = 0.043, P = 0.039, NES = 2), which contained 25 core genes (ABCC1, GCLC, NDUFB4, TXNRD1, SOD2, FTL, PRDX1, PRNP, SRXN1, GLRX, GSR, PRDX6, GCLM, NDUFA6, GPX4, SOD1, HHEX, GPX3, PFKP, MGST1, TXN, GLRX2, ATOX1, FES, and MPO). The expression (**Figure 7D**) and prognosis (**Figure 7E**) of the core genes of the reactive oxygen pathway and 20 genes predicted by the GeneMANIA database in liver cancer were analyzed using the GSCA database. Later, the Venn diagram (**Figure 7F**) revealed that SCIN could play a role in liver cancer through ABCC 1, CAPG and PRNP, and the relationship between the three genes and SCIN in liver cancer was verified in GSCA database (**Figure 7G**).

### 3.8 Suppression of SCIN reduces cell proliferation and tumor growth in LIHC bearing mice

**Figure 8:**
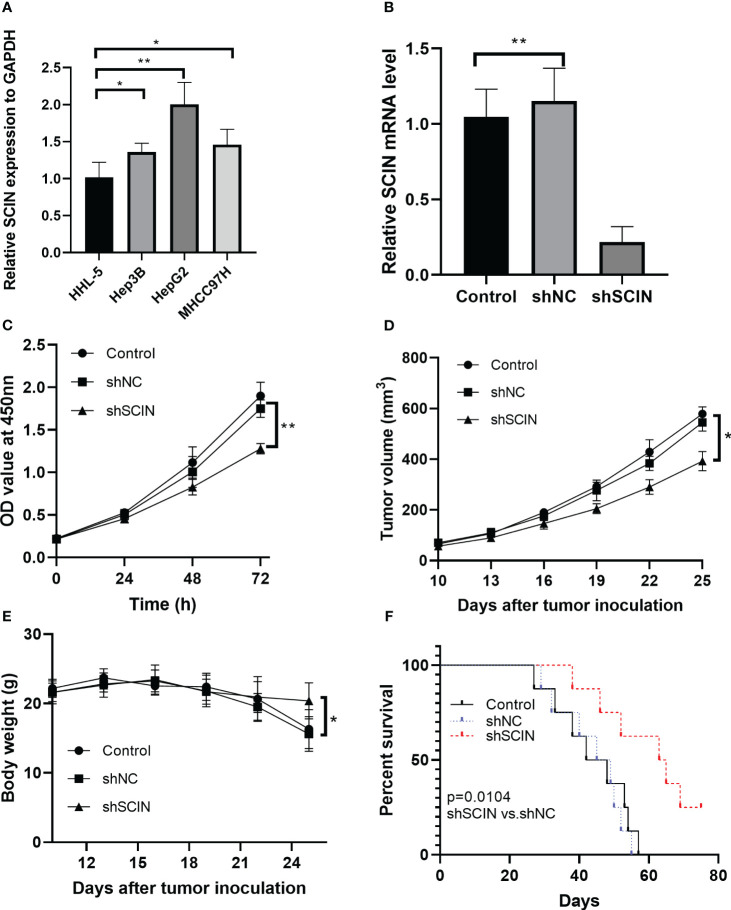
SCIN knockdown affects cell proliferation and tumor growth in tumor bearing mice. **(A)** mRNA expression of SCIN was detected in hepatocellular carcinoma cell lines. **(B)** SCIN was knocked down in HepG2 cells by adenoviral vectors carrying sh-SCIN. **(C)** Cell viability was analyzed with CCK8 assay. **(D)** Tumor bearing mice were treated with HepG2 LV-shSCIN cells or control cells. Tumor volume was monitored in each group. **(E)** The body weight of mice was recorded. **(F)** Survival curve of mice in control, sh-SCIN and sh-NC group. *p<0.05, **p<0.01.

The overexpression of SCIN was determined in hepatocellular carcinoma cell lines, compared with normal control cells (**Figure 8A**). Since there was a highest expression of SCIN in HepG2 cells, we used this cell line for further analysis. To dissect the role of SCIN in LIHC, SCIN was silenced in HepG2 cells by adenoviral vectors carrying sh-SCIN. The high-efficiency shRNA mediated SCIN knockdown was verified by RT-qPCR analysis (**Figure 8B**). SCIN knockdown cells elicited obvious declined cell viability by comparison with the controls (**Figure 8C**). Given that SCIN knockdown reduced tumor cell proliferation, we next investigated whether the suppression of SCIN was associated with reduced tumor growth. **Figure 8D** illustrated that the tumor growth diminished in mice subcutaneously inoculated with HepG2 LV-shSCIN cells, whereas the tumors grew progressively in mice injected with HepG2 LV-shNC cells or HepG2 cells (**Figure 8E**). To address the impact of SCIN knockdown on survival time, the survival rate of experimental animals was recorded post tumor cells inoculation. Expectably, the suppression of SCIN prolonged survival time of mice models (**Figure 8F**). Collectively, SCIN knockdown could inhibit the hepatocellular carcinoma growth and enhance survival rate of tumor bearing mice.

## 4 Discussion

Pan-cancer analysis is a widely used bioinformatics method for cancer investigation, with the advances of providing promising insights for early prevention and effective treatment of cancer (22, 23). Pan-cancer analysis can increase statistical power and facilitate the identification of new patterns of genomic biomarkers (24). The analysis of genomic changes and functions in various cancer lineages can provide the opportunity for discovering the common treatment method for the similar phenotype of the cancers (25). Numerous studies strongly suggested that SCIN was highly expressed in a series of cancers, and exerted promising prognostic value for pan-cancer (6–11). Jian et al. reported that SCIN was overexpressed in breast cancer tissues, and played an oncogenic role in the pathogenesis of breast cancer pathogenesis (26). The similar expression pattern of SCIN was found in glioma and the SCIN was correlated with the poor prognosis (27). For the aberrant expression of SCIN and its association with cell metastasis and poor prognosis, SCIN has been proposed as the therapeutic target of many types of cancers, such as CRC (28) and glioma (27). Although the specific role of SCIN has been identified in single tumor type, the detailed role of SCIN and related mechanism in pan-cancer has not been elucidated. To our knowledge, this is the first comprehensive study of the biological function of SCIN from a pan-cancer perspective. According to our research, it is found that SCIN exerts non-negligible role in the progress and prognosis of various cancers, especially in LIHC.

Pan-cancer differential expression analysis suggested SCIN expression was significantly increased in CHOL, LIHC, LUAD, and PRAD, but decreased in COAD, HNSC, KIRC, LUSC, and STAD compared with adjacent normal tissues. It is reported that SCIN is a novel oncogene that facilitates the proliferation and suppresses apoptosis of breast cancer cells (26). Another study has suggested that the expression of SCIN inhibits the proliferation and tumor formation of human megakaryoblastic leukemia cells in nude mice (29). Thus, SCIN plays dual roles in different cancer types. Further analysis revealed that the SCIN transcription level was correlated with lymph node metastasis of PRAD, and was related to the pathological stage of KIRC and LIHC. Similarly, the previous evidence showed that SCIN expression was correlated with lymph node metastasis and pathological stage of CRC (28). These findings revealed the significant role of SCIN in the development of cancers. Additionally, SCIN overexpression was related to the worse prognosis in KICH, LGG, LIHC, and UCEC, whereas SCIN de-expression was related to poor DSS in KIRC and KIRP. The survival analysis showed that SCIN-high expression was correlated with poor disease free survival of CRC patients (28), which was in agreement with our validation analysis in LIHC. All these mentioned above revealed SCIN exerted distinct role in different cancer types.

Accumulating evidences have demonstrated that SCIN not only promoted the metastasis of gastric cancer (6–8), lung cancer (10), liver cancer (11), and glioma (28), but also inhibited tumor progression in prostate cancer (9) and breast cancer (26), showing that SCIN has promoting and suppressive effects in tumors.

It is reported that SCIN and Cofilin−1 (CLF1) are the members of the actin-binding protein family, both of which are overexpressed in breast cancer and significantly correlated with tumor stage and lymph node metastasis (30). As a function effector of tumor metastasis suppressor factor (BRMS1), SCIN plays a regulatory role in the apoptosis of hepatocellular carcinoma cells (11). Thus, addressing the regulation or coordination of proteins in cell−motility machinery may provide novel insight to understand the dual role of SCIN in different cancer types. It is generally believed that cancer is caused by genetic mutations, which confers biological resistance of tumor cells, so genetic mutation can increase the risk for tumor occurrence and development (31). Our data suggested that the highest frequency of alteration in SCIN (5.29%) was seen in patients with UCEC, in which “mutation” was the predominant type, while “amplification” was the predominant type of genetic alteration in bladder urothelial cancer, with a frequency of about 3.65%, manifesting as missense mutations. Further, our results indicated that SCIN mutations significantly impacted OS (P = 6.013e-03), PFS (P = 0.0121), and DSS (P = 1.248e-03) of Sarcoma patients. Thus, genetic alteration of SCIN may affect substrates expressions, however the role of SCIN mutations in tumor tissues is rarely studied. Next, in our research, relationships between SCIN and MMRs genes, MSI, TMB, and DNA methyltransferases, as well as neoantigen were assessed. In comparison to normal tissues, SCIN protein expression in various tumor tissues is related to the above indicators, which may explain the distinct regulatory role of SCIN in different cancers. Further investigation on the association between SCIN expression and mutation levels is required.

According to a comprehensive analysis, SCIN was found to be highly expressed and correlated with survival, immune cell infiltration, immune checkpoint gene, immune subtype, molecular subtype, MMRs, and DNA methylation in LIHC. The GSEA analysis in our study showed that the SCIN-related genes were significantly enriched in reactive oxygen species pathway. Reactive oxygen species (ROS) are the production of metabolism, which are implicated in various signal transduction pathways and biological activities, including immune response. It is reported that ROS plays a critical role in tumor microenvironment by exerting effects on immune cells (32). ROS can be produced by tumor cells and immune infiltrated cells, which is associated with immunosuppressive response (33). ROS accumulation reduced the T cell recognition and T cell immune response in tumor microenvironment (34). The function of tumor infiltrating lymphocytes was impaired by ROS production. Thus, SCIN may affect survival, and immune microenvironment by regulating ROS pathway. Furthermore, we performed network analyses to identify the key SCIN-related genes related to LIHC. ABCC1 (r = 0.549, P = 8.34e-31), CAPG (r = 0.384, P = 1.47e-14), and PRNP (r =0.345, P = 7.04e-12) that significantly correlated with SCIN were identified. As one of the ATP-binding cassette superfamily of transporters, ABCC1 is expressed in several tissues, including the liver, kidneys, intestine, and brain (35, 36). One of the previous investigations determined the overexpression of ABCC1 in clinical HCC samples. Additionally, ABCC1 was strongly correlated with macrophages, which was served as the biomarker for HCC prognosis and response to therapy (37). CAPG belonging to gelsolin protein family, is widely scattered in cytoplasm and nucleus (38, 39). Compared to normal counterparts, HCC specimens displayed increased CAPG expression and the overexpression of CAPG was correlated with greater mortality. Additionally, CAPG expression was positively associated with migration and invasive ability of HCC cell lines (40). PRNP is a Cuproptosis-related gene, which exerts function in regulating apoptosis, cellular signaling and cell migration (38–42). It is reported that PRNP could be served as the immune therapy target for HCC (43). Our experiments *in vitro* determined the abnormal upregulation of SCIN in hepatocellular carcinoma cells. To further dissect the role of SCIN in LIHC, we knocked down the SCIN expression in hepatocellular carcinoma cells. Results indicated that SCIN knockdown suppressed the proliferative ability of hepatocellular carcinoma cells. A previous study revealed that SCIN suppression was related with the inhibited proliferation of human prostate cancer (44). Liu et al. also reported the SCIN silencing showed inhibitive effect on lung cancer cell proliferation (10). The previous findings above were in agreement with our results *in vivo*. The pervious evidence determined the prognosis predictive role in gastric cancer (6). To verify the prognostic role of SCIN in LIHC, we constructed tumor-bearing animal models and treated by lentivirus-mediated silencing of SCIN cells. The results showed that SCIN knockdown enhanced survival rate of tumor bearing mice. Together, we demonstrate that SCIN plays an important role in mediating the growth process of liver cancer.

In our study, SCIN showed significant correlation with multiple immune cells, immune subtypes, and immune checkpoint genes in different malignancies, including LIHC. In LIHC cohort, high expression of SCIN and lower scores of basophils (P = 3e-4), CD4+T cells (P = 0.00037), CD8+ cells (P = 0.00041), eosinophils (P = 0.00035), mesenchymal stem cells (P = 2e-04), type 2 T helper cell scores (P = 0.0069) and higher scores of regulatory T cell scores (P = 0.00071) were related with the worst OS. It means that SCIN might regulate and recruit immune cells to promote or inhibit the progression of cancers, and thus play a complexly regulative role in tumor progression. Additionally, Wang’ study revealed that SCIN expression was correlated with immune infiltration, and outcomes of glioma patients (27). Overall, SCIN expression may affect LIHC development and prognosis through regulating immune infiltration. However, the mechanisms behind these interactions require further investigation.

With regard to the clinical relevance of SCIN with tumor stage and prognosis and its key roles in cell growth *in vivo* and *in vitro*, SCIN elicits the potential to be used as the biomarker for the prognosis and development of liver cancer. Consistently, SCIN has been proposed as the potential target in different tumor types, such as CRC (28) and prostate cancer (9). However, some limitations of this study should be acknowledged. First, the aberrant expression of SCIN in liver cancer were not validated in a large number of clinical samples. Secondly, our study did not explore the detailed mechanisms underlying the role of SCIN involved in cancer development. Thus, studies with large clinical samples and experimental models are warranted to validate our findings. In addition, the deep investigation of the correlation between SCIN with its relevant genes and immune cell infiltration may shed light on understanding the dual role of SCIN in different cancer types and provide evidence of SCIN as the therapeutic target for cancers.

## 5 Conclusions

Above all, our study systematically and comprehensively summarized the role of SCIN in pan-cancer, especially in LIHC. SCIN may promote tumor occurrence and subsequent progression of various cancers though DNA methylation, mutation, and tumor immune cell infiltration. Aberrant expression of SCIN promoted hepatocellular carcinoma cells proliferation partly *in vivo* and *in vitro*, and SCIN silence prolonged the OS in animal models to some extent. Our research provides novel insights into the mechanism of SCIN expression in pan-cancer and improve the possibility of the SCIN target therapy in LIHC.

## Data availability statement

The original contributions presented in the study are included in the article/**Supplementary Material**. Further inquiries can be directed to the corresponding author.

## Ethics statement

The animal study was approved by The Animal Care and Ethics Committee of Weifang People’s Hospital. The study was conducted in accordance with the local legislation and institutional requirements.

## Author contributions

SZ: Conceptualization, Data curation, Formal Analysis, Writing – original draft. YL: Formal Analysis, Methodology, Resources, Writing – review & editing. YY: Formal Analysis, Resources, Writing – review & editing. WL: Conceptualization, Methodology, Writing – review & editing. XL: Conceptualization, Investigation, Writing – review & editing. KL: Investigation, Resources, Writing – review & editing. JQ: Conceptualization, Investigation, Writing – review & editing. LZ: Conceptualization, Investigation, Project administration, Supervision, Writing – review & editing.

## Supplementary material

Supplementary Figure 1 The heatmap of the correlation between infiltrated immune cells with SCIN in different cancer types. *P < 0.05, **P < 0.01, ***P < 0.001, ****P < 0.0001.

Supplementary Figure 2 Venn diagram of the comparison of data in TCGA, GEPIA2, Kaplan-Meier database, SCIN expression.
