# Supplementary figures and images for: Scinderin is a potential prognostic biomarker and correlated with immunological regulation: from pan-cancer analysis to liver hepatocellular carcinoma
Source: Front Immunol. 2024 Jul 23;15:1361657. doi: 10.3389/fimmu.2024.1361657 (PMC11300247; doi:10.3389/fimmu.2024.1361657)

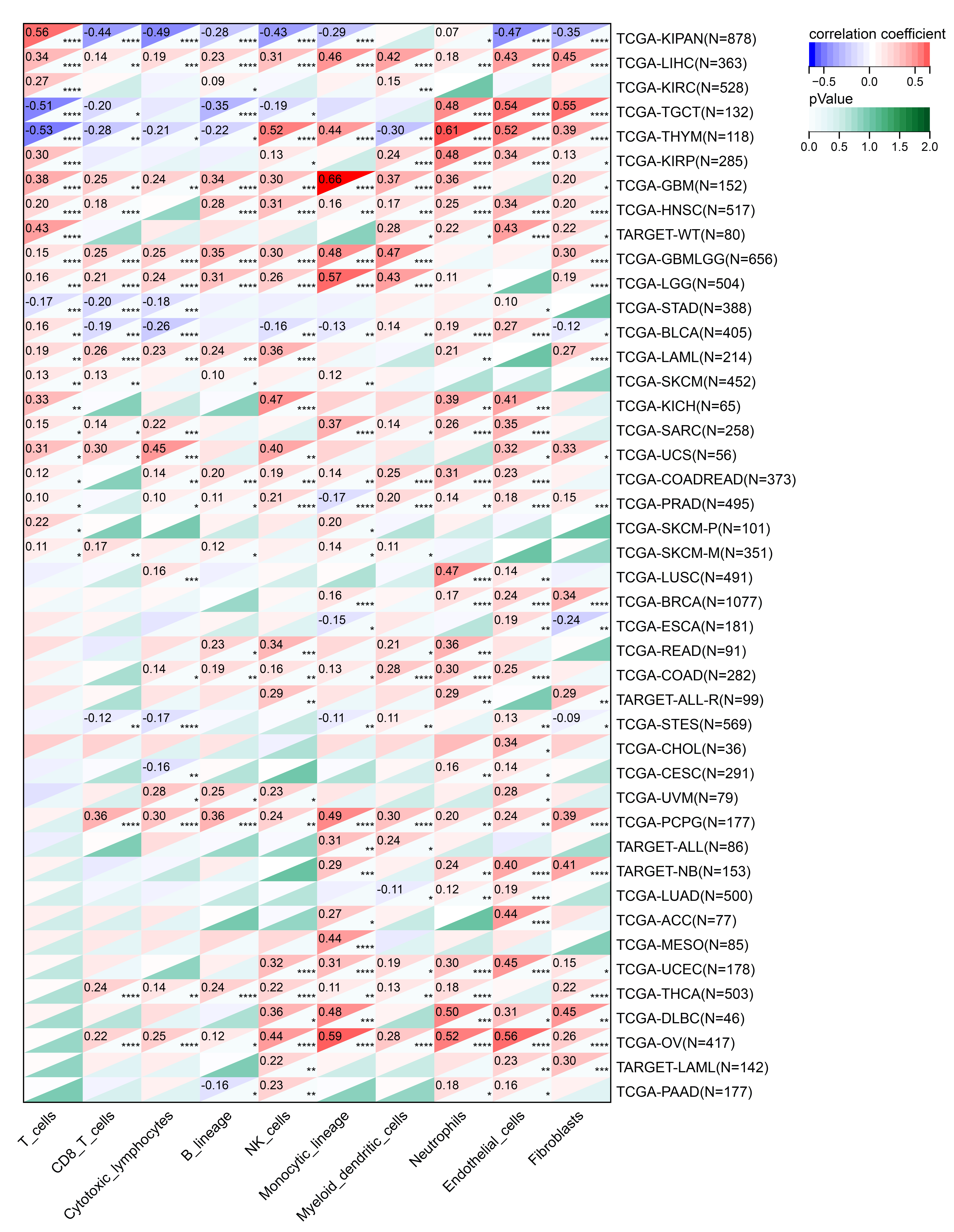

Supplement: Supplementary Figure 1 — The heatmap of the correlation between infiltrated immune cells with SCIN in different cancer types. *P < 0.05, **P < 0.01, ***P < 0.001, ****P < 0.0001. [file Image_1.tif]

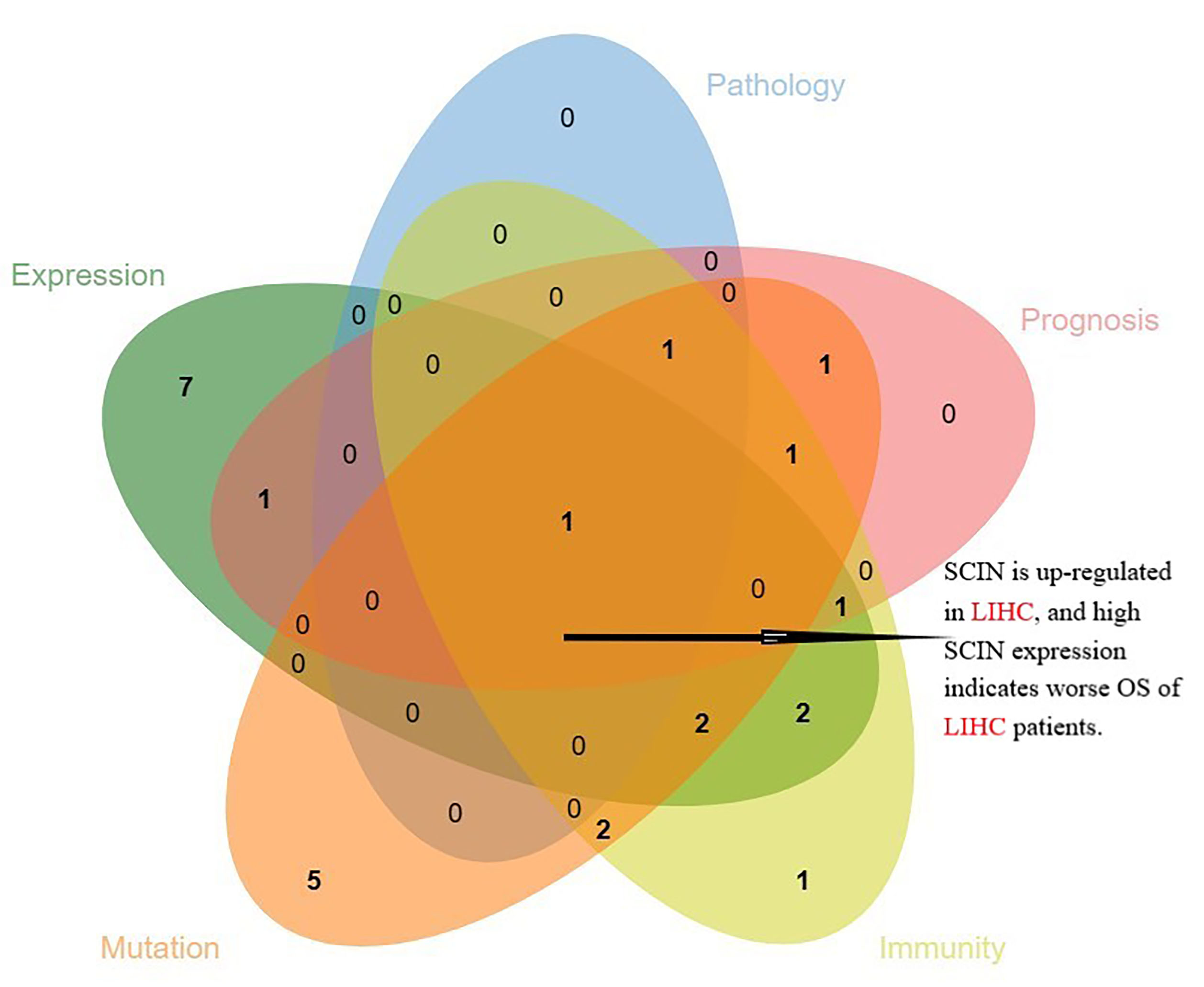

Supplement: Supplementary Figure 2 — Venn diagram of the comparison of data in TCGA, GEPIA2, Kaplan-Meier database, SCIN expression. [file Image_2.tif]
